# B7-H3 molecular imaging for the era of pan-cancer targeted therapy

**DOI:** 10.3389/fphar.2026.1923470

**Published:** 2026-10-06

**Authors:** Baoyan Liu, Kelin Wang, Kewen He, Yong Wang

**Affiliations:** 1 Department of Occupational Pulmonology, Shandong First Medical University Affiliated Occupational Disease Hospital (Shandong Occupational Disease Hospital), Jinan, Shandong, China; 2 Department of Radiation Oncology, Shandong Cancer Hospital and Institute, Shandong First Medical University and Shandong Academy of Medical Sciences, Jinan, Shandong, China

**Keywords:** affibody, B7-H3, bicyclic peptide, CD276, immunoPET, PET/CT, radioimmunotherapy, theranostics

## Abstract

B7-H3 (CD276) is broadly overexpressed across solid tumors and plays active roles in immune evasion, tumor angiogenesis, and metabolic reprogramming. As B7-H3-directed antibody–drug conjugates chimeric antigen receptor T-cell therapies, and radioimmunotherapy agents advance through clinical trials, non-invasive whole-body imaging of B7-H3 expression has become a pressing clinical need. Tissue biopsy with IHC cannot capture systemic target heterogeneity, and the recent IDeate-PanTumor01 trial found no correlation between baseline B7-H3 H-score and response to ifinatamab deruxtecan—illustrating the gap that molecular imaging must fill. This mini review surveys B7-H3-targeted imaging agents by scaffold class—antibodies, affibodies, nanobodies, and bicyclic peptides—and examines radioimmunotherapy platforms that couple PET-based dosimetry with targeted radionuclide delivery.

## 1 Introduction

B7-H3, also designated CD276, is a type I transmembrane glycoprotein of the B7 immune checkpoint superfamily. In humans, B7-H3 is expressed on cell membranes as two distinct transmembrane isoforms resulting from exon duplication and alternative splicing: a predominant four-immunoglobulin domain form (4Ig-B7-H3; duplicated tandem IgV–IgC architecture) that represents the dominant membrane species in human solid tumors, and a shorter two-immunoglobulin domain form (2Ig-B7-H3; single IgV–IgC pair), whereas rodents express only the 2Ig isoform (Mielcarska et al., 2025; Larkin et al., 2025; Guo et al., 2025; Zhao et al., 2025). In addition, a soluble species (sB7-H3/s2Ig) detectable in the circulation and cerebrospinal fluid is generated either through alternative splicing yielding secretory variants or via proteolytic ectodomain shedding of membrane B7-H3 mediated by matrix metalloproteinases (MMPs) and ADAM family disintegrins (Digregorio et al., 2021; Ma et al., 2025). Crucially, the preferential expression of B7-H3 protein in malignancies is governed predominantly at the post-transcriptional level: while *CD276 mRNA* is broadly transcribed across normal human tissues, physiological protein translation is stringently silenced by microRNAs targeting the 3′-untranslated region, notably the miR-29 family (miR-29a/b/c) (Liu et al., 2021). In tumor tissues, widespread downregulation of miR-29 and oncogenic activation trigger selective translational derepression, driving robust B7-H3 cell-surface protein overexpression on malignant cells, cancer-associated fibroblasts, and neoangiogenic vasculature while sparing normal host parenchyma (Corrigan et al., 2025; Picard et al., 2026; Abdelfattah et al., 2026). Beyond B7-H3, recent advances across the broader B7 immune checkpoint family—such as novel B7-H6-targeted bispecific antibodies and IL-15 fusion platforms engineered to eradicate chemo-resistant solid tumors (Ma et al., 2025)—further underscore the therapeutic momentum in this field and accentuate the shared clinical need for non-invasive companion imaging to guide patient stratification. Zhai et al. catalogued 830 publications on CD276 in cancer from 2001 to August 2025 (21.77% annual growth), confirming that the field has shifted decisively from mechanistic biology toward clinical therapeutics (Zhai et al., 2026). ADC programs targeting B7-H3 have now reached Phase 3, and over twenty clinical trials evaluating B7-H3-directed CAR-T cell therapies are actively underway worldwide (Favours et al., 2026; Brown et al., 2025; Monaca et al., 2026).

Against this backdrop, identifying which patients express B7-H3 — and where—has become critical (Kontos et al., 2021). IHC on a single biopsy is limited by intratumoral heterogeneity: Xia et al. showed that B7-H3 expression differs between tumor cells, intratumoral stroma, and peritumoral tissue within the same lesion, and that high stromal expression can mislead treatment evaluation (Xia et al., 2025). Although circulating soluble B7-H3 has been hypothesized to act as a potential competitive sink, definitive clinical evidence demonstrating significant reduction of *in vivo* tumor uptake remains pending. More importantly, because certain malignancies—such as gliomas and pediatric neuroepithelial tumors—can co-express both membranous 4Ig and 2Ig isoforms (Digregorio et al., 2021; Ma et al., 2025), developing pan-isoform radiotracers capable of recognizing both membrane-bound species represents a vital design strategy to maximize diagnostic sensitivity and eliminate false-negative evaluations (Glazer et al., 2026). PET/CT with a specific B7-H3 radiotracer offers a non-invasive, whole-body map of target expression, bridging the critical gap between localized lesion biophysics and systemic therapeutic deployment (Wang Y. et al., 2026; Wang et al., 2025a; Assadi et al., 2026). The following sections review what has been achieved with each class of probe.

## 2 Antibody-based probes and theranostic ADCs

Full-length antibodies (∼150 kDa) were the first B7-H3 imaging scaffolds explored for nuclear imaging (Liu et al., 2021; Ahmed et al., 2015; Xu et al., 2009). Burvenich et al. labeled the anti-B7-H3 antibody DS-5573a with ^89^Zr and demonstrated tumor-specific PET uptake in xenograft models; the probe required 5–7 days post-injection to achieve adequate tumor-to-background ratios owing to slow blood-pool clearance (Burvenich et al., 2018). The anti-B7-H3 antibody MGD009, which drives T-cell-redirected killing via a DART bispecific format, was subsequently labeled with ^89^Zr for imaging of T-cell co-regulator engagement, further expanding the antibody-based imaging toolkit (Nagase-Zembutsu et al., 2016; Liu et al., 2025). Zheng et al. developed [^89^Zr]Zr-DFO-hu4G4, a recombinant humanized antibody probe targeting the extracellular domain of B7-H3 (Zheng et al., 2024). Radiochemical purity exceeded 99% with a specific activity of 74 MBq/mg, and the tracer retained >90% purity after 7 days in serum, outperforming the reference probe [^89^Zr]Zr-DFO-DS-5573a in head-to-head xenograft comparisons. These studies collectively illustrate that ^89^Zr-immuno-PET can delineate B7-H3 expression with high specificity, but the fundamental pharmacokinetic limitation of full-length antibodies—slow clearance requiring multi-day imaging windows—limits their practical utility for routine clinical PET.

A more nuanced pharmacokinetic picture emerged from Fernandes et al., who tracked two ^89^Zr-labeled anti-B7-H3 antibodies longitudinally in xenograft models (Fernandes et al., 2025). One probe ([^89^Zr]Zr-Ab-1) showed high initial tumor uptake but unexpectedly decreasing retention over time, attributed to proteolytic cleavage by matrix metalloproteinases (MMPs) within the tumor microenvironment—a mechanism with implications for dosimetry planning in radioligand therapy. The second probe demonstrated more stable retention, consistent with preserved epitope access. These findings highlight how target biology and the tumor microenvironment can override molecular weight as the dominant determinant of antibody pharmacokinetics, a consideration relevant to probe design for all immunoglobulin-based scaffolds.

To avoid ambiguity between conventional antibody–drug conjugates (ADCs) and radioimmunotherapy (RIT) agents, we specifically designate dual-action constructs bearing both a chemotherapeutic payload and a radiometal chelator as 'radiolabeled ADCs'. Qiu et al. constructed such a theranostic platform from a B7-H3-targeting ADC radiolabeled with either ^89^Zr or ^177^Lu. They first confirmed by IHC that B7-H3 expression in prostate cancer primary tumors and bone metastases was high and, strikingly, remained stable after androgen ablation, chemotherapy, and radiotherapy—a contrast with PSMA, which can be downregulated under therapy. In 22RV1 xenografts, [^89^Zr]Zr-B7-H3 ADC accumulated to 33.2 ± 1.0 %ID/g at 144 h, with tumor-to-muscle ratios exceeding 15. Therapeutically, integrated [^177^Lu]Lu-B7-H3 ADC + radionuclide therapy outperformed radiolabeled antibody alone, ADC monotherapy, and sequential treatment without increasing organ toxicity; hematological changes were transient and reversible (Qiu et al., 2026).

Parallel work from Liang et al. developed a matched theranostic pair — [^89^Zr]Zr-DFO-B7-H3mab for immuno-PET and [^177^Lu]Lu-DOTA-B7-H3mab for RIT—in Huh7 hepatocellular carcinoma xenografts. Both probes achieved radiochemical purity >99%, with PET uptake of 2.47 ± 0.41 %ID/g at 192 h in B7-H3-positive tumors; the 11.1 MBq therapeutic dose achieved 80.2% ± 17.6% tumor growth inhibition at day 19 with minimal hematological toxicity (Liang et al., 2026). Together with the prostate cancer ADC data from Qiu et al., these results argue that B7-H3 expression stability across treatment lines confers a meaningful advantage over targets subject to therapy-induced downregulation.

A more targeted approach to the isoform problem was reported by Glazer et al. They developed MIL33B, an IgG2a antibody with high affinity for the tumor-associated 4Ig-B7-H3 (*K*_D_ = 7.23 × 10^−11^ M) and 8- to 18-fold selectivity over the soluble 2Ig form (*K*_D_ = 4.11 × 10^−8^ M); binding to PD-L1, PD-L2, PD-1, CTLA-4, B7-H2, and B7-H4 was below the limit of detection. [^89^Zr]Zr-DFO-MIL33B showed significantly higher blood-pool-normalized tumor retention in 4Ig-B7-H3-expressing HeLa (3.25 ± 0.65 vs. 0.79 ± 0.13 in KO cells, *p* = 0.0201) and CT26 syngeneic models compared with the isotype control. In the immunocompetent CT26 model, a single systemic dose of [^90^Y]Y-MIL33B (100 µCi) achieved 53% long-term survival; immunophenotyping confirmed CD8^+^ T-cell activation and durable immunological memory, demonstrating that isoform-selective beta-radioligand therapy can prime systemic anti-tumor immunity (Glazer et al., 2026). This study establishes isoform selectivity as a design criterion with both imaging and therapeutic consequences: probes that cannot distinguish 4Ig-B7-H3 from the circulating 2Ig decoy may underestimate true lesion uptake or suffer competitive inhibition at the target site.

## 3 Small engineered scaffolds: affibodies and beyond

### 3.1 Affibody molecules

Affibody molecules (∼7 kDa, 58 amino acids core) are non-immunoglobulin scaffold proteins selected from a combinatorial library based on a three-helix bundle domain derived from staphylococcal protein A (Tolmachev and Orlova, 2020; Eissler et al., 2024; Da Pieve and Kramer-Marek, 2024; Wang H. et al., 2025). Their small size enables rapid blood clearance within 1–2 h, permitting same-day radiotracer administration and imaging—a decisive practical advantage over antibody-based probes (Eissler et al., 2024; Da Pieve and Kramer-Marek, 2024). The development of B7-H3-targeted affibody molecules traces a coherent evolutionary trajectory across four distinct stages: initial proof-of-concept discovery, affinity maturation and dimerization, radiometal/chelator optimization, and first-in-human clinical PET validation (Supplementary Figure S1).

Oroujeni et al. first demonstrated B7-H3-targeted radionuclide imaging using the high-affinity affibody AC12 (*K*_D_ = 0.9 ± 0.6 nM) labeled with ^99m^Tc via a C-terminal GGGC chelator, obtaining specific SPECT images in xenograft models with a tumor-to-blood ratio of 11.0 ± 0.5 already at 4 h post-injection (Oroujeni et al., 2022). Notably, this ratio already exceeded the 5.03 ± 0.73 achieved by ^89^Zr-DS-5573a at 7 days, illustrating the kinetic advantage of small scaffolds for diagnostic imaging.

Recognizing that SPECT sensitivity and spatial resolution are inferior to PET, the Uppsala group pursued systematic optimization of the B7-H3 affibody series toward ^68^Ga-based PET agents. Oroujeni et al. performed affinity maturation of AC12, identifying variants with substantially improved binding compared with the parental sequence (Oroujeni et al., 2023). A parallel strategy of dimerization was explored in Oroujeni et al., who compared homodimeric ZAC12*-ZAC12*-GGGC (EC_50_ = 1.5 nM vs. SKOV-3 cells) and monovalent bi-affibody ZAC12*-ZTaq_3-GGGC (EC_50_ = 73.6 nM) with the affinity-matured monomer SYNT-179 (EC_50_ = 8.1 nM) (Oroujeni et al., 2024). The homodimer achieved the tightest apparent binding through avidity, confirming that dimerization can compensate for moderate intrinsic affinity; however, the size increase associated with dimerization must be weighed against the potential for slower tumor clearance.

Tolmachev et al. compared ^99m^Tc-GGGC and ^111^In-DOTA labeling of the same affibody backbones (SYNT179 and AC12). Despite higher binding affinity *in vitro*, ^111^In-labeled variants achieved significantly greater tumor uptake in SKOV-3 xenografts—tumor-to-blood ratio of 31.1 ± 2.9 for [^111^In]In-SYNT179-DOTA vs. 15.6 for the ^99m^Tc counterpart—attributed to the residualizing property of the DOTA/^111^In label, though at the cost of higher renal retention (Tolmachev et al., 2025a). These results confirm that radionuclide and chelator choice independently modulate *in vivo* imaging contrast beyond intrinsic affinity.

Moving to PET with ^68^Ga, Tolmachev et al. evaluated four affibody variants (ZB7-H3_2, ZB7-H3_3, ZB7-H3_4, and ZAC12 as control) conjugated with NOTA for ^68^Ga labeling. Among these, ZB7-H3_2 showed the most favorable tumor-to-organ ratios in SKOV-3 xenografts, providing the first direct evidence that ^68^Ga-labeled affibody molecules can achieve high-contrast B7-H3 PET imaging at 1–2 h post-injection (Tolmachev et al., 2025b). These preclinical data directly informed the chelation chemistry comparison described below.

Labeling chemistry was the focus of Tolmachev et al., who compared the acyclic THP chelator with conventional cyclic NOTA for ^68^Ga-labeling of affibody ZB7-H3_2. THP achieved 100% radiochemical yield at room temperature in 5 min without a post-labeling purification step; NOTA required 60 °C heating with lower yield. *In vitro* binding affinity (IC_50_: 2.6 ± 0.4 vs. 3.2 ± 0.5 nM, *p* > 0.05) and *in vivo* SKOV-3 xenograft uptake were statistically equivalent between the two conjugates (Tolmachev et al., 2026). For clinical translation, the room-temperature, single-step, kit-based THP protocol—analogous to the procedures established for clinical ^68^Ga-PSMA and ^68^Ga-DOTATATE kits—has a decisive practical advantage in terms of GMP compliance and radiopharmacy workflow.

Xia et al. reported [^68^Ga]Ga-B7H3-BCH, an AC12-derived affibody with a RESCA chelator (*K*_D_ = 4.5 nM) (Xia et al., 2025). In a prospective clinical study (NCT06454955, n = 20 patients, 10 tumor types), this probe detected 51 of 60 lesions (85.0%) versus 49 of 60 by ^18^F-FDG (81.7%); nine lesions were B7H3-BCH-positive but FDG-negative, including eight metastatic sites. Using an SUV_max_ cutoff of 3.85, the probe detected B7-H3 IHC positivity with 84.38% sensitivity and 100% specificity (AUC 0.9707). Optimal imaging time was 50–60 min post-injection based on dynamic PET kinetics. A limitation was high physiological kidney and liver uptake—the authors proposed albumin-binding modifications or PEG extension to address this—consistent with the renal accumulation pattern observed across the affibody class owing to glomerular filtration and tubular reabsorption of small, cationic proteins. This first-in-human dataset marks the clinical culmination of this stepwise affibody optimization cascade, positioning [^68^Ga]Ga-B7H3-BCH as the most clinically advanced B7-H3 PET probe to date (Supplementary Figure S1).

### 3.2 Nanobodies and bicyclic peptides

Nanobodies (∼15 kDa) offer a middle-ground pharmacokinetic profile (Sheng et al., 2025; Yuan et al., 2025; Eftekhari and Kazemi-Lomedasht, 2026). Funeh et al. immunized llamas with recombinant human B7-H3 and, following phage-display biopanning, identified single-domain antibodies (sdAbs) displaying nanomolar affinities and promiscuous cross-reactivity to both human 4Ig and 2Ig isoforms as well as murine B7-H3 (Funeh et al., 2025). The lead candidate, [^99m^Tc]Tc-C51, was evaluated by micro-SPECT/CT at 1.5 h post-injection in two subcutaneous xenograft models (U87-MG glioblastoma and 624MEL melanoma). The probe demonstrated high-contrast tumor visualization with tumor uptake reaching 4.96 ± 1.40 %IA/g and an outstanding tumor-to-blood ratio of 10.0 ± 3.16 at 1.5 h, with binding specificity confirmed via cold blocking (50-fold excess) and flow cytometry.

Peptide-based tracers remain the least explored class but carry advantages in synthetic accessibility and chemical tunability (Wang et al., 2025c; Shabsigh and Solomon, 2024; Diao et al., 2026). Zhang et al. synthesized three ^68^Ga-DOTA-bicyclic peptide probes (FZ1/FZ2/FZ3) from a scaffold first identified with *K*_D_ = 43.5 nM, differing only in PEG linker length (0, 2, or 4 units). All showed radiochemical purity >96%. FZ1 (no PEG spacer) had the best affinity (*K*_D_ = 83.22 nM) and tumor uptake (3.50 ± 0.97 %ID/g at 30 min in B7-H3-overexpressing H1299 tumors, versus 1.09 ± 0.18 %ID/g in low-expressing controls). Uptake correlated positively with B7-H3 protein levels across six xenograft models (*R* ^2^ = 0.7816, *p* < 0.0001). Renal uptake increased steeply with PEG length (FZ1: 13.37 ± 2.34 to FZ3: 30.2 ± 4.81 %ID/g), identifying linker optimization as the key next development step (Zhang et al., 2026). The physicochemical and pharmacokinetic diversity of B7-H3-targeting radiolabeled probes—spanning intact antibodies, isoform-selective immunoglobulins, theranostic ADCs, affibody molecules, nanobodies, and bicyclic peptides—together with the clinical radioimmunotherapy agents and the B7-H3-directed ADC discussed below, is comprehensively summarized in Table 1. A streamlined head-to-head comparison highlighting the trade-offs, advantages, and limitations across these scaffold classes is provided in Supplementary Table S1.

**TABLE 1:** B7-H3- targeted molecular imaging probes and radioligand therapy agents: comprehensive summary.

Probe (scaffold)	Isotope	Binding affinity	Modality	Study stage	Key data (from primary literature)	Source
^89^Zr-DS-5573a (intact mAb, ∼150 kDa)	^89^Zr	nM range (Scatchard analysis)	ImmunoPET	Preclinical (MDA-MB-231 xenograft)	Tumor uptake: 32.32 ± 6.55 %ID/g at day 7 post-injection; immunoreactivity day 0: 75.0% ± 2.9%; serum stability day 7: 57.2% ± 2.0%; requires 5–7-day imaging window due to slow blood-pool clearance	(Burvenich et al., 2018)
[^89^Zr]Zr-DFO-hu4G4 (recombinant humanized mAb)	^89^Zr	IC_50_ = 0.75 ± 0.83 nM (DFO-hu4G4 vs. B7-H3, U87 cells)	ImmunoPET	Preclinical (CT26-CD276/GL261-CD276 xenografts)	RCP >99%; specific activity 74 MBq/mg; serum stability >90% at 168 h; tumor uptake 22.57 ± 3.03 %ID/g at 48 h (CT26-CD276); T/M ratio 27.65 ± 3.17 vs. KO 11.68 ± 4.19 (*p* < 0.001); biodistribution comparable to [^89^Zr]Zr-DFO-DS-5573a (no significant difference)	(Zheng et al., 2024)
[^89^Zr]Zr-DFO-MIL33B (4Ig-isoform-selective IgG2a)	^89^Zr/90Y	*K*_D_ = 7.23 × 10^−11^ M (human 4Ig-B7-H3); 8× selectivity over human 2Ig; >18× over murine 2Ig; binding to PD-L1, PD-1, CTLA-4, B7-H2, B7-H4: below detection limit	ImmunoPET + β-RLT	Preclinical (HeLa xenograft; CT26 syngeneic immunocompetent model)	^89^Zr-PET: blood-pool-normalized tumor retention 3.25 ± 0.65 vs. 0.79 ± 0.13 in B7-H3 KO HeLa (*p* = 0.0201). Single systemic dose^90^Y-MIL33B (100 µCi): 53% long-term survival in CT26 syngeneic model; immunophenotyping confirmed CD8^+^ T-cell activation and durable immunological memory	(Glazer et al., 2026)
[^89^Zr]Zr-DFO/[^177^Lu]Lu-DOTA-B7-H3 ADC (theranostic ADC platform)	^89^Zr/^177^Lu	*K*_D_(ADC) = 15.1 nM *K*_D_(Ab) = 171.8 nM (BLI)	ImmunoPET + RLT	Preclinical (22RV1 prostate cancer xenograft)	IHC confirmed high, stable B7-H3 expression in primary tumors and bone metastases after androgen ablation, chemotherapy, and radiotherapy. [^89^Zr]Zr-B7-H3 ADC tumor uptake: 33.2 ± 1.0 %ID/g at 144 h; T/muscle >15. Integrated [^177^Lu]Lu-ADC + RLT outperformed radiolabeled antibody alone, ADC monotherapy, and sequential treatment; hematological changes transient and reversible	(Qiu et al., 2026)
[^89^Zr]Zr-DFO-B7-H3mab/[^177^Lu]Lu-DOTA-B7-H3mab (mAb, hepatocellular carcinoma)	^89^Zr/177Lu	*K*_d_ = 221.2 ± 119.9 nmol/L ([^177^Lu]Lu-DOTA-B7-H3mab vs. Huh-7 cells; radioligand binding assay)	ImmunoPET + RIT	Preclinical (Huh-7 HCC subcutaneous xenograft)	RCP >99% for both probes (radio-iTLC); stable >99% in saline/10% FBS for 10 days^89^Zr-PET: tumor SUVmean 2.47 ± 0.41 at 192 h p.i. (n = 3).^177^Lu biodistribution: tumor uptake 5.86 ± 2.26 → 12.58 ± 2.42 %ID/g (day 1→day 4).^177^Lu-DOTA-B7-H3mab (11.1 MBq): tumor growth inhibition 80.2% ± 17.6% at day 19 (n = 8); H&E and hematological analyses confirmed minimal toxicity	(Liang et al., 2026)
[^99m^Tc]Tc-AC12-GGGC (affibody, ∼7 kDa)	^99m^Tc	*K*_D_1 = 1.9 ± 0.8 nM (weight = 23%); *K*_D_2 = 68.8 ± 7.4 nM (weight = 67%) (LigandTracer, living SKOV-3 cells)	SPECT	Preclinical (SKOV-3 xenograft)	Proof-of-concept for B7-H3-targeted radionuclide imaging with small scaffold proteins. Tumor-to-blood ratio: 11.0 ± 0.5 at 4 h p.i. — superior to^89^Zr-DS-5573a at day 7 (5.03 ± 0.73), demonstrating the pharmacokinetic advantage of the affibody scaffold	(Xia et al., 2025; Oroujeni et al., 2022; Tolmachev et al., 2026)
[^99m^Tc]Tc-SYNT179-GGGC vs. [^111^In]In-SYNT179-DOTA (affinity-matured affibody; non-residualizing vs. residualizing label)	^99m^Tc/^111^In	[^99m^Tc]Tc-SYNT179: *K*_D_1 = 0.028 ± 0.001 nM (weight = 12%); *K*_D_2 = 8.2 ± 0.5 nM (weight = 88%). [^111^In]In-SYNT179-DOTA: *K*_D_1 = 0.06 ± 0.04 nM (weight = 20%); *K*_D_2 = 13 ± 2 nM (weight = 80%) (LigandTracer, living SKOV-3 cells)	SPECT	Preclinical (SKOV-3 xenograft)	^111^In-DOTA (residualizing): tumor uptake 3.63 ± 0.31 %ID/g at 4 h — significantly higher than^99m^Tc-GGGC (1.59 ± 0.19 %ID/g; *p* < 0.05). Tumor-to-blood: 31.09 ± 2.9 (^111^In) vs. 15.74 ± 4.13 (^99m^Tc).^111^In renal retention ∼30× higher; hepatobiliary excretion lower. Best imaging window: 3–4 h p.i	(Tolmachev et al., 2025a)
[^99m^Tc]Tc-ZAC12*-ZAC12*-GGGC (homodimeric affibody; dimerization vs. affinity maturation study)	^99m^Tc	*K*_D_1 = 0.28 ± 0.10 nM (weight = 68%; avidity, LigandTracer); EC_50_ = 1.5 nM (SKOV-3, flow cytometry)	SPECT	Preclinical (SKOV-3 xenograft)	Despite higher apparent avidity, tumor uptake 1.25 ± 0.36 %ID/g at 4 h — not significantly different from affinity-matured monomer SYNT-179 (2.17 ± 0.54 %ID/g; *p* > 0.05) and not superior to parental ZAC12-GGGC (1.04 ± 0.08 %ID/g). Conclusion: dimerization does not compensate for the size-induced reduction in tumor extravasation rate	(Oroujeni et al., 2024)
[^68^Ga]Ga-NOTA-ZB7-H3_2 (affibody; optimal variant from 4-candidate screen)	^68^Ga	EC_50_ = 7.3 nM (SKOV-3, flow cytometry); SPR confirmed improved binding vs. ZAC12 (EC_50_ = 57.1 nM)	PET/CT	Preclinical (SKOV-3 xenograft)	RCP 99.9% ± 0.2%; stable in EDTA challenge and PBS at 2 h. Tumor uptake: 8.96 ± 1.16 %ID/g at 2 h–2.8-fold higher than control ZAC12 (3.24 ± 0.77 %ID/g; *p* < 0.05). Tumor-to-liver ratio: 7.5 ± 0.7 (best among 4 variants). Tumor-to-blood ratio 1.5× higher than best reported^99m^Tc-affibody. nanoPET/CT confirmed B7-H3-mediated tumor uptake *in vivo*	(Tolmachev et al., 2025b)
[^68^Ga]Ga-THP-Z_B7-H3_2_ vs. [^68^Ga]Ga-NOTA-Z_B7-H3_2_ (affibody; chelator comparison for clinical translation)	^68^Ga	IC_50_: THP = 2.6 ± 0.4 nM; NOTA = 3.2 ± 0.5 nM (*p* > 0.05; *in vitro* equivalent)	PET	Preclinical (SKOV-3 xenograft)	THP: 100% radiochemical yield at room temperature in 5 min; no post-labeling purification required. NOTA: requires 60 °C heating with lower yield. Tumor uptake statistically equivalent (*p* > 0.05). NOTA-ZB7-H3_2 showed slightly higher tumor-to-liver ratio (p < 0.05). THP protocol enables kit-based, GMP-compliant radiolabeling analogous to established^68^Ga-PSMA/DOTATATE kits	(Tolmachev et al., 2026)
[^68^Ga]Ga-B7H3-BCH (AC12-derived affibody; RESCA chelator)	^68^Ga	*K*_D_ = 4.5 nM (SPR)	PET/CT	Phase 1 clinical (NCT06454955; n = 20 patients; 10 tumor types)	Lesion detection: 51/60 (85.0%) vs.^18^F-FDG 49/60 (81.7%); 9 FDG-negative/BCH-positive lesions (8 metastatic sites). At SUV_max_ cutoff 3.85: sensitivity 84.38%, specificity 100%, AUC 0.9707. Optimal imaging window: 50–60 min p.i. (dynamic PET kinetics). High physiological renal and hepatic uptake noted; albumin-binding modification or PEG extension proposed	(Xia et al., 2025)
^99m^Tc-C51 nanobody (∼15 kDa; phage-display selected from immunized llama library)	^99m^Tc	Nanomolar range (flow cytometry); pan-isoform cross-reactive (human 4Ig, human 2Ig, and murine B7-H3)	SPECT	Preclinical (two xenograft tumor models)	Identified by phage-display screening against B7-H3-immunized llamas; high-contrast micro-SPECT/CT images; favorable biodistribution; cross-reactive with murine B7-H3 (enables direct human/murine B7-H3 comparison in the same animal). Nanobody scaffold supports Fc-fusion, PEGylation, or albumin-binding domain engineering to modulate pharmacokinetics	(Funeh et al., 2025)
[^68^Ga]Ga-DOTA-B7H3-FZ1/2/3 (bicyclic peptide, ∼2.2 kDa; PEG linker 0/2/4 units; FZ1 = optimal variant)	^68^Ga	*K*_D_(FZ1) = 83.22 nM (BLI, probe vs. B7-H3 protein); Parental scaffold: *K*_D_ = 43.5 nM	Micro-PET/CT	Preclinical (6 xenograft models; cross- model validation)	RCP >96% (all three probes). FZ1 tumor uptake: 3.50 ± 0.97 %ID/g at 30 min (B7-H3-OE H1299) vs. 1.09 ± 0.18 %ID/g (low-expressing control). Uptake positively correlated with B7-H3 protein levels across 6 models (*R* ^2^ = 0.7816; *p* < 0.0001). Renal uptake increased steeply with PEG length: FZ1 13.37 ± 2.34 → FZ3 30.2 ± 4.81 %ID/g. No acute toxicity observed	(Zhang et al., 2026; Bowler et al., 2023)
[^124^I]-8H9 (intact mAb; intrapontine CED; DIPG-specific delivery)	^124^I (theranostic)	—	PET dosimetry + RIT	Phase 1 clinical (NCT01502917; n = 28 pediatric DIPG patients; 7 dose cohorts)	No DLT across all cohorts (9.25–148 MBq); 2 grade-3 AEs (1 hemiparesis, 1 skin infection). Mean Vd/Vi = 3.4 (SD 1.2); mean lesion absorbed dose = 0.39 Gy/MBq; lesion-to-whole-body dose ratio >1200 — a therapeutic index unachievable by systemic delivery	(Souweidane et al., 2018)
[^131^I]-omburtamab (intact mAb; intraventricular Ommaya reservoir delivery)	^131^I	—	SPECT dosimetry + compartmental RIT (cRIT)	Phase 1 clinical (n = 38, CNS malignancies; 100 injections; 370–2960 MBq)	RP2D = 1850 MBq. CSF absorbed dose: 1.01 mGy/MBq vs. blood 0.04 mGy/MBq (CSF-to-blood ratio ∼25); all major organs below tolerance. Grade 3/4 thrombocytopenia: predominant hematological toxicity. Neuroblastoma subgroup (cRIT): median PFS = 7.5 years	(Kramer et al., 2022)
Ifinatamab deruxtecan (I-DXd) (B7-H3-directed ADC; DXd chemotherapy payload)	— (chemotherapy payload)	—	Therapeutic ADC (companion imaging gap identified)	Phase 1/2 clinical (NCT04145622; n = 97; 10 tumor types)	RP2D = 12.0 mg/kg (MTD not reached). Confirmed ORR at ≥4.8 mg/kg (n = 70): 34% (95% CI 23–47). SCLC subgroup (n = 21, ≥6.4 mg/kg): ORR 52%, median PFS 5.6 months (95% CI 3.9–8.1), median OS 12.2 months. Drug-related ILD: 5% (1 fatal event at 16.0 mg/kg). Critically: no correlation between baseline B7-H3 H-score and tumor shrinkage — IHC alone cannot identify the therapeutically relevant patient population	(Johnson et al., 2026)

## 4 Radioimmunotherapy with B7-H3 antibodies

Two clinical programs have used B7-H3-targeting antibodies as vehicles for compartmental radionuclide delivery in CNS tumors. Souweidane et al. conducted a phase 1 dose-escalation trial of [^124^I]-8H9 delivered directly into the pons via convection-enhanced delivery (CED) in 28 children with diffuse intrinsic pontine glioma (DIPG). The theranostic radionuclide ^124^I was chosen because its positron emission enables PET-based dosimetry while its beta/gamma emission provides therapeutic effect. No dose-limiting toxicity was found across seven cohorts (9.25–148 MBq); only two patients had grade 3 adverse events (1 hemiparesis, one skin infection). Mean Vd/Vi ratio was 3.4 (SD 1.2), mean lesion absorbed dose 0.39 Gy/MBq, and the lesion-to-whole-body dose ratio exceeded 1200 — a therapeutic index unachievable by systemic delivery. The trial continues with expanded enrolment (NCT01502917) (Souweidane et al., 2018).

Kramer et al. reported a phase 1 study of intraventricular ^131^I-omburtamab—the same anti-B7-H3 antibody radioiodinated for compartmental delivery into the CSF via Ommaya reservoir—in 38 patients with CNS malignancies (16 neuroblastoma, 22 other solid tumors; 100 injections; 370–2960 MBq). The recommended phase 2 dose was 1850 MBq. Dosimetry confirmed a CSF-to-blood absorbed dose ratio of ∼25 (1.01 vs. 0.04 mGy/MBq), with major organs consistently below tolerance. Grade 3/4 thrombocytopenia was the predominant hematological toxicity. In the neuroblastoma subgroup receiving cRIT, median progression-free survival was 7.5 years—a result that underscores the potential of B7-H3-directed locoregional RIT in a disease with otherwise dismal prognosis (Kramer et al., 2022). These clinical dosimetry findings carry profound implications for companion PET probe design and theranostic development. The dramatic ∼25-fold CSF-to-blood dose advantage of ^131^I-omburtamab and the >1200:1 lesion-to-whole-body dose ratio achieved with convection-enhanced ^124^I-8H9 demonstrate that pre-therapeutic PET dosimetry using matched diagnostic positron emitters (such as ^124^I paired with ^131^I, or ^89^Zr paired with ^177^Lu) is essential to verify actual tracer infusion volume (V_d_/V_i_), assess tissue permeability, and prospectively select patients prior to high-activity therapeutic intervention. Furthermore, comparing compartmental delivery with the systemic theranostic [^89^Zr]Zr-/[^177^Lu]Lu-B7-H3 ADC platform developed by Qiu et al. (Qiu et al., 2026) reveals that while locoregional delivery circumvents systemic clearance sinks in CNS tumors, systemic theranostics requires precise immunoPET guidance to balance tumor accumulation against off-target bone marrow retention. Thus, quantitative PET-based dosimetry serves as a universal navigational tool across both compartmental RIT and systemic radioligand therapies to optimize the therapeutic index.

## 5 Clinical ADC results and the imaging gap

The urgency for companion B7-H3 imaging was sharpened by Johnson et al., who reported dose-escalation data from IDeate-PanTumor01 (NCT04145622), a first-in-human phase 1/2 trial of ifinatamab deruxtecan (I-DXd) in 97 patients across 10 tumor types. The maximum tolerated dose was not reached; the recommended dose for expansion was 12.0 mg/kg. The confirmed objective response rate among 70 efficacy-evaluable patients at ≥4.8 mg/kg was 34% (95% CI 23–47). In the post-hoc small-cell lung cancer subgroup (n = 21 at ≥6.4 mg/kg), ORR was 52% (95% CI 30–74), median PFS 5.6 months (95% CI 3.9–8.1), and median OS 12.2 months. Drug-related interstitial lung disease occurred in five patients (5%), with one fatal event at 16.0 mg/kg. B7-H3 expression testing was not required for enrolment, and—critically—no correlation was observed between baseline B7-H3 IHC H-score and tumor shrinkage in the SCLC subgroup, exposing a profound diagnostic gap (Johnson et al., 2026). Mechanistically, we propose that whole-body PET succeeds where static IHC fails because the two modalities measure fundamentally different biological phenomena. While IHC quantifies total antigen density in a fixed, permeabilized tissue section—a static *ex vivo* measurement that cannot differentiate intracellular pools from cell-surface receptors or assess vascular patency—PET with a validated radiotracer directly interrogates *in vivo* target accessibility. That is, immunoPET quantifies the actual fraction of B7-H3 that is spatially reachable and bindable by circulating macromolecules under the tumor’s physiological microenvironment, which is often governed by abnormal perfusion, dense desmoplasia, and elevated interstitial fluid pressure. Furthermore, whole-body PET circumvents the severe spatial sampling bias of single-lesion biopsies by mapping systemic inter- and intra-metastatic target heterogeneity across the entire disease burden (Fraum et al., 2024; Sunderland et al., 2025; Providencia et al., 2026). A probe’s affinity and isoform selectivity further modulate this predictive fidelity: a high-affinity tracer selectively targeting the tumor-associated 4Ig-B7-H3 isoform (such as [^89^Zr]Zr-DFO-MIL33B (Glazer et al., 2026)) bypasses potential sequestration by circulating soluble s2Ig decoys and specifically interrogates the tumor-cell membrane pool, providing an authentic functional surrogate for ADC payload delivery. Although this isoform-specific predictive hypothesis warrants prospective validation in companion clinical trials, non-invasive PET imaging clearly bridges the gap between localized histopathology and systemic targeted pharmacology. To fill this diagnostic and therapeutic gap, the clinical translation of B7-H3-targeted probes must balance pharmacokinetic clearance, tissue penetration, and tumor retention across diverse molecular scaffolds, ranging from systemic intact immunoglobulins to rapid-kinetics small engineered platforms (Figure 1).

**FIGURE 1:**
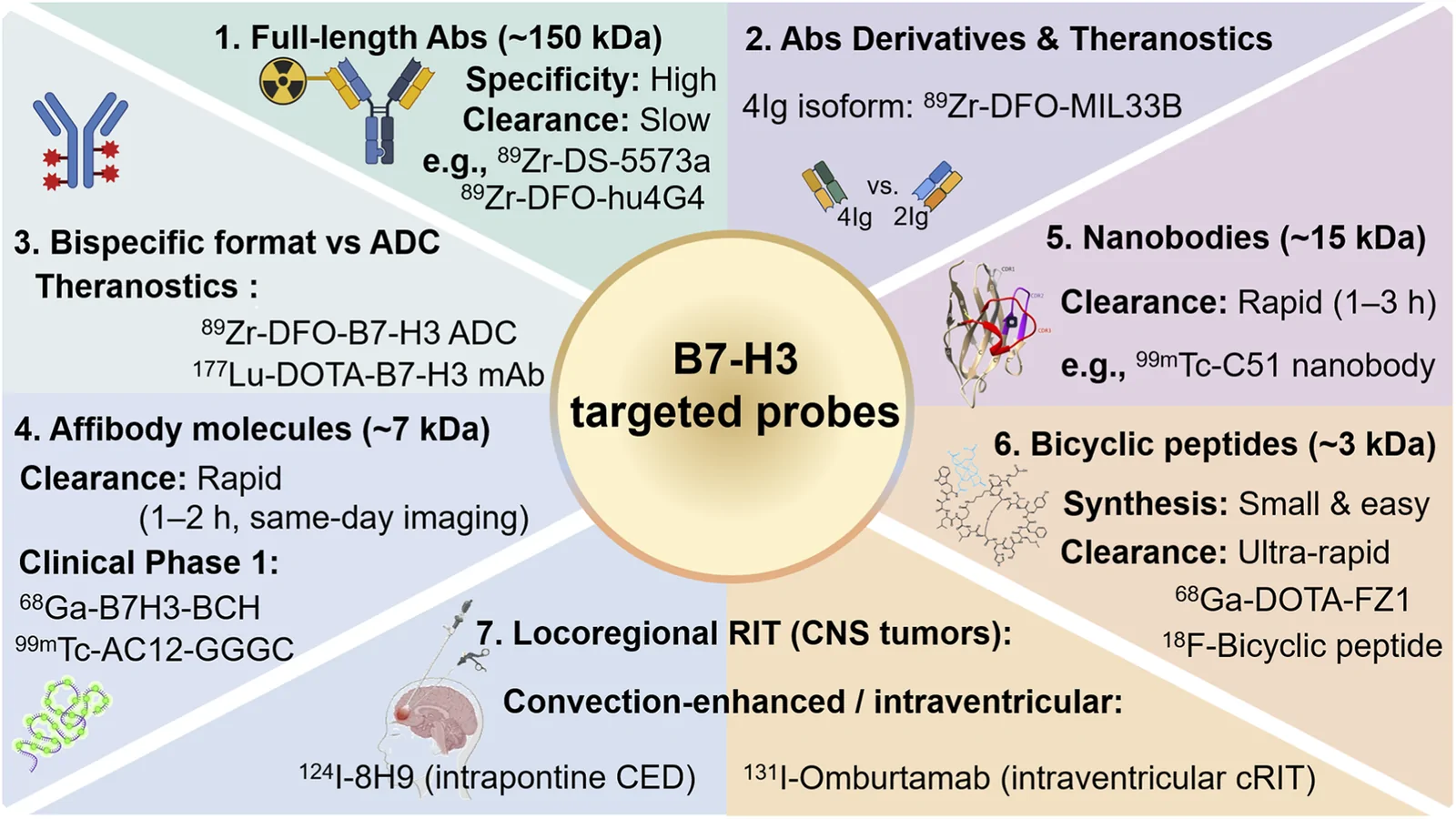
Structural diversity and theranostic applications of B7-H3-targeted molecular imaging probes.

## 6 Discussion

The field has moved from proof-of-concept SPECT to first-in-human PET/CT within roughly 5 years of identifying high-affinity B7-H3 binding scaffolds—a pace driven by the clinical urgency of companion diagnostics for a wave of B7-H3-directed therapeutics (Wang J. et al., 2026; McAteer et al., 2025). Several themes cut across all probe classes reviewed here.

Intratumoral heterogeneity and the two-isoform problem are the central biological challenges. Xia et al. showed that B7-H3 distribution across tumor cells, stroma, and peritumoral tissue varies considerably within individual lesions (Xia et al., 2025), and Glazer et al. demonstrated that the circulating s2Ig-B7-H3 decoy can competitively absorb imaging agents that lack isoform selectivity (Glazer et al., 2026). MIL33B’s picomolar affinity for the tumor-associated 4Ig form with orders-of-magnitude selectivity over the 2Ig form offers a conceptual solution, though its slow antibody-scale pharmacokinetics still require multi-day imaging windows.

Across scaffold classes, the pharmacokinetic trade-off is intimately linked to TME-level physical and enzymatic barriers. While full-length antibodies and ADCs provide prolonged tumor retention essential for therapeutic dosimetry, their slow convective transport renders them susceptible to perivascular arrest under elevated interstitial fluid pressure (IFP) and proteolytic degradation by tumor-derived MMPs over multi-day circulation windows (Fernandes et al., 2025). Conversely, small engineered scaffolds (affibodies ∼7 kDa, nanobodies ∼15 kDa, and bicyclic peptides ∼2–3 kDa) rapidly diffuse across dense stroma, effectively bypassing high IFP and achieving peak tumor-to-background contrast within 1–2 h before extensive enzymatic cleavage can occur. From a scaffold-engineering perspective, overcoming TME barriers will require: (i) incorporating protease-resistant linkers and framework rigidification to prevent premature tracer cleavage in aggressive microenvironments; (ii) optimizing molecular charge and PEGylation to decouple rapid interstitial penetration from renal cortical retention; and (iii) balancing 4Ig-isoform selectivity against pan-isoform binding to navigate circulating soluble decoys versus membranous target heterogeneity (Zhang et al., 2026). The clinical implementation of kit-formulated THP chelation (Tolmachev et al., 2026) already addresses practical radiopharmacy hurdles, setting the benchmark for small-scaffold optimization.

While this review has spotlighted ADCs and RIT due to their direct theranostic synergy and mature companion imaging literature, the clinical imperative for whole-body B7-H3 PET extends with equal force to CAR-T cells, bispecific T-cell engagers (BiTEs/DARTs, e.g., MGD009), and checkpoint-blocking antibodies (e.g., enoblituzumab) (Favours et al., 2026; Brown et al., 2025; Monaca et al., 2026). Because productive T-cell redirecting therapies and CAR-T cytotoxicity strictly depend on exceeding specific cell-surface antigen-density thresholds to form stable immunological synapses, static biopsy IHC cannot reliably predict whole-body immune engagement. Non-invasive PET quantification of real-time, functional B7-H3 receptor density thus provides a crucial biomarker to guide patient enrollment and monitor antigen down-modulation across all B7-H3-directed immunotherapeutic platforms.

The radioimmunotherapy data from Souweidane et al. (Souweidane et al., 2018) and Kramer et al. (Kramer et al., 2022) have shown that B7-H3-targeted compartmental RIT is both safe and clinically meaningful in pediatric CNS tumors, with therapeutic indices that systemic delivery cannot replicate. The prostate cancer theranostic ADC platform from Qiu et al. (Qiu et al., 2026) shows that this concept extends to systemic disease when B7-H3 expression is stable under therapeutic pressure—a property that distinguishes it from PSMA. The ifinatamab deruxtecan data (Johnson et al., 2026) make clear that a companion imaging tool is needed: enrollment based on anatomical criteria alone left responders and non-responders mixed together. Prospective integration of B7-H3 PET into ADC and CAR-T trials is the logical next step.

## 7 Conclusion

B7-H3-targeted molecular imaging has transitioned from preclinical proof-of-concept to early clinical feasibility (Oroujeni et al., 2023; Souweidane et al., 2018). However, rather than an imminent clinical tool, B7-H3 PET currently represents a highly promising candidate companion modality whose ultimate clinical utility depends on rigorous, prospective biomarker-driven validation (Glazer et al., 2026; Johnson et al., 2026). To bridge the gap between early clinical imaging and therapeutic decision-making, we envision a clear translational roadmap for future clinical trial designs: (i) integrating baseline whole-body [^68^Ga]Ga-B7H3-BCH PET/CT to quantify systemic target accessibility using established metrics (such as the SUVmax cutoff of 3.85 and total functional B7-H3 tumor volume); (ii) stratifying patients into PET-positive and PET-negative/borderline cohorts within ADC (e.g., ifinatamab deruxtecan) or CAR-T trials to prospectively determine whether *in vivo* PET avidity outperforms baseline tissue IHC in predicting therapeutic response; (iii) implementing longitudinal on-treatment PET scans to assess whether early uptake reductions reflect on-target response or target down-modulation and immune escape; and (iv) standardizing room-temperature, kit-based chelator platforms (such as acyclic THP) to enable decentralized GMP production and facilitate multi-center adoption. Systematically executing these validation steps will establish whether whole-body B7-H3 PET can definitively overcome the spatial limitations of static histology and serve as an indispensable companion diagnostic for precision oncology.
