# Supplementary material for: B7-H3 molecular imaging for the era of pan-cancer targeted therapy
Source: Front Pharmacol. 2026 Oct 6;17:1923470. doi: 10.3389/fphar.2026.1923470 (PMC13680445; doi:10.3389/fphar.2026.1923470)
Supplement: Supplementary file 1 [file Table1.docx]

**Supplementary Table S1. Head-to-Head Comparative Summary of B7-H3-Targeted Molecular Imaging Scaffold Classes**

| **Scaffold Class** | **Approx. MW** | **Primary Clearance Route** | **Optimal Imaging Window** | **Key Pharmacokinetic & Clinical Advantages** | **Main Translational & Technical Limitations** | **Representative Tracers** |
| --- | --- | --- | --- | --- | --- | --- |
| **Full-length mAbs** | ~150 kDa | Hepatic / RES | 48–168 h (2–7 days) | • Excellent overall tumor uptake & retention • Matched pharmacokinetics for ADC/RIT dosimetry | • Slow blood clearance (high background) • Requires long-lived isotopes (^89^Zr) • Susceptible to circulating s2Ig decoy sink | [^89^Zr]Zr-DS-5573a [^89^Zr]Zr-DFO-hu4G4 [^89^Zr]Zr-DFO-MIL33B |
| **Affibody Molecules** | ~7 kDa | Renal filtration | 1–2 h (Same day) | • Rapid extravasation & high early contrast • Compatible with single-step kit labeling (THP) • Clinically validated ([^68^Ga]Ga-B7H3-BCH) | • High physiological renal & hepatic retention • Glomerular filtration requires mitigation strategies | [^68^Ga]Ga-B7H3-BCH [^68^Ga]Ga-THP-Z_B7-H3_2_ [^99m^Tc]Tc-AC12-GGGC |
| **Nanobodies (VHH)** | ~15 kDa | Renal filtration | 1–3 h (Same day) | • Intermediate kinetics balancing contrast & tumor retention • High stability & modular engineering capacity | • Renal uptake requires tail modifications • Modest liver uptake depending on framework charge | ^99m^Tc-C51 nanobody |
| **Bicyclic Peptides** | ~2–3 kDa | Rapid renal excretion | 0.5–1 h (Same day) | • Extremely rapid tissue penetration & clearance • Fully synthetic production without cell culture | • Lower intrinsic affinity (*K*_D_ > 40 nM) • Short tumor residence time requires affinity maturation | [^68^Ga]Ga-DOTA-B7H3-FZ1 |
